# Effect of Coordinating Impurities on the Electrochemical Stability of Polymeric Nickel(II) Schiff-Base Complexes

**DOI:** 10.3390/ijms27041685

**Published:** 2026-02-09

**Authors:** Ulyana M. Rodionova, Daniil A. Lukyanov, Peixia Yang, Ruopeng Li, Oleg V. Levin, Elena V. Alekseeva

**Affiliations:** 1 Institute of Chemistry, Saint-Petersburg University, 199034 Saint-Petersburg, Russia; 2 MIIT Key Laboratory of Critical Materials, Technology for New Energy Conversion and Storage, School of Chemistry and Chemical Engineering, Harbin Institute of Technology, Harbin 150001, China

**Keywords:** nickel(II) Salen-type polymer, Schiff base, electrochemical stability, supercapacitor, lithium-ion battery, methanol, isopropanol

## Abstract

Polymer films of nickel Schiff-base complexes were investigated to clarify degradation mechanisms induced by coordinating impurities—specifically, the protic solvents methanol and isopropanol. Films of poly[Ni(Salen)] and its sterically protected derivatives were electropolymerized in situ and subjected to cyclic voltammetry (CV) and electrochemical quartz crystal microbalance (EQCM) measurements in dry acetonitrile electrolyte with 1% vol. alcohol added. In situ monitoring of redox activity and mass changes revealed something. It was revealed that traces of alcohols act as axial ligands to the Ni center. This disrupts the conjugated π-system and conductivity of the polymer. The rate of electrochemical stability strongly depends on the complex structure. The unsubstituted poly[Ni(Salen)] film showed the fastest loss of capacity in both methanol and isopropanol, whereas complexes with methyl substituents in the diimine bridge (poly[Ni(Salpn-1,2)] and poly[Ni(Saltmen)]) exhibited significantly improved stability. EQCM measurements revealed irreversible changes in the mass of all polymer films upon exposure to alcohol-containing electrolytes. These observations are consistent with the axial coordination of alcohol molecules to the Ni centers and the concomitant ingress of solvent species into the polymer matrix. The results demonstrate that molecular design—specifically, introducing steric hindrance around the metal center—markedly enhances resistance to coordinating impurities.

## 1. Introduction

Electrochemical capacitors (supercapacitors) are a promising energy-storage technology, especially for the rapidly growing markets of electric vehicles and portable electronics. Supercapacitors offer high power density (fast charge/discharge), long cycle life (up to 10^6^ cycles), and a relatively low temperature sensitivity [1,2]. However, their energy density is still lower than that of batteries, which motivates strategies to increase stored energy either by enhancing electrode material capacitance or by widening the operating voltage via electrolyte and active material optimization [3,4]. Both strategies can be effectively achieved by using conducting polymers, which offer a unique combination of high electronic conductivity [5], capacitance [6], and broad electrochemical stability [7]. A wide range of conducting polymers, including polypyrrole [8], polyaniline [9,10], polythiophene [11], and their composites with carbon nanotubes [12], graphene [13], or metal oxides, have been systematically investigated as electrode materials for supercapacitor applications [14].

Among these advanced materials, polymeric transition-metal complexes with Schiff-base ligands have attracted considerable attention as electrode materials for supercapacitors and related electrochemical devices [15,16,17,18]. Such metal–Salen complexes exhibit high electroactivity, good redox reversibility, and cyclic stability [17,19,20,21,22]. A wide variety of transition-metal ions (Ni, Cu, Co, Pd, Mn, Fe, etc.) can form Salen-type complexes, allowing their electrochemical properties to be tuned over a broad range [18,23,24,25,26,27,28]. Nickel(Salen) polymers in particular have shown exceptional performance in supercapacitor electrodes, achieving specific capacitances on the order of 70–75 F·g^−1^ at low charge–discharge rates [15,25,29,30,31,32,33]. These metallopolymers incorporate multiple redox-active centers and promise high performance in energy-storage applications, including supercapacitors and lithium-ion batteries [25]. Reviews of the field underscore that polymers obtained via Schiff-base coordination chemistry represent a versatile class of redox-active materials for sustainable battery and supercapacitor electrodes [15,27,32].

Despite their attractive electrochemical properties, a serious limitation for the practical application of metal–Salen polymers is their sensitivity to coordinating impurities such as water in non-aqueous electrolytes [34,35,36,37,38,39]. Even trace amounts of moisture can coordinate to the Ni center of Ni(Salen) polymers and trigger the rapid degradation of electrochemical activity, especially in the oxidized state. Recent studies have demonstrated that the degradation of Ni–Salen films in the presence of water begins with axial coordination of a water molecule to the Ni(II) center, which disrupts the conjugated π-system and sharply decreases electronic conductivity. Incorporating substituents such as methyl or methoxy groups into the ligand structure hinders coordination to the metal center and promotes peripheral interactions with water, thereby improving the structural and electrochemical stability of the complex [34]. For example, the poly[Ni(Saltmen)] polymer, which contains four methyl groups on the diimine bridge (providing strong steric hindrance around the metal center), shows the greatest stability in electrolytes, containing ~1% H_2_O, whereas analogous polymers with fewer or no bridge substituents degrade much faster [35]. In situ conductivity and spectroscopic measurements confirm that water coordination forces the Ni–Salen complex from a square-planar to an octahedral geometry, breaking the intrachain charge transport pathways. DFT calculations further indicate that water binding is thermodynamically favorable for all complexes, but in sterically protected complexes, the kinetic access of H_2_O to the Ni center is impeded, yielding a much more robust structure. Thus, steric factors rather than electronic factors predominantly determine a complex’s resistance to axial ligand coordination by impurities [35].

While the deleterious impact of water on Ni–Salen polymer stability has been studied in detail [29,34,35,36,38,40], the effect of other common contaminants of organic solvents in non-aqueous electrolytes (e.g., monoalcohols) remains insufficiently explored. Such species can also act as axial ligands and potentially induce degradation via a similar mechanism. In real-world operating conditions of supercapacitor and battery systems, organic solvent impurities like methanol or isopropanol may be present as residual solvents after standard electrode processing or components of hybrid electrolytes [41]. Even small concentrations of organic impurities in an electrolyte can significantly affect its properties. These considerations highlight the importance of understanding how impurities like alcohols influence electrode material stability.

In this context, the present work focuses on the influence of electrolyte impurities (methanol and isopropanol) on the electrochemical stability of Ni(II) Salen-type metallopolymer cathode materials. These two alcohols were selected due to the contrast in steric bulk of their hydroxyl-bearing groups (methanol being small and isopropanol being more hindered), which was expected to influence their coordination behavior with the Ni center. We investigate a series of Ni Schiff-base polymer films with varying ligand structures (unsubstituted vs. methyl-substituted) to evaluate how steric ligand design impacts stability in the presence of coordinating alcohols. In this work, the alcohol concentration of 1 vol.% was deliberately chosen as a stress-test condition to simulate unfavorable electrolyte purity scenarios, such as the presence of residual solvents after electrode processing or electrolyte preparation. Axial coordination of protic ligands to the oxidized Ni center in Ni(Salen)-type polymers is thermodynamically favorable and can disrupt the conjugated polymer backbone, leading to the degradation of electronic conductivity. Consequently, even trace levels of coordinating impurities are expected to initiate similar degradation pathways, although at significantly slower rates. A similar stress-test strategy was previously employed to investigate the effect of water impurities, where degradation trends observed at elevated water concentrations (1 vol.% and above) were found to correlate well with those obtained in the presence of trace moisture levels [34,35]. This demonstrated that accelerated testing at higher impurity content provides reliable insight into degradation mechanisms relevant under practical operating conditions. Therefore, the behavior observed at elevated impurity concentration represents an upper limit of degradation severity, while the same qualitative trends are anticipated at lower impurity levels relevant to practical systems.

CV and EQCM methods are employed simultaneously to monitor the electrochemical response and mass changes in the polymer films, providing insight into the molecular-level mechanisms of film degradation. The combined CV/EQCM data were analyzed to distinguish different mass-transfer processes: a reversible ion exchange (insertion/extraction of charge-compensating anions) typically yields a two-step mass vs. charge response for Ni(Salen) polymers in dry electrolytes, while any axial coordination of alcohol molecules should manifest as an additional continuous mass increase during oxidation cycles (since coordinated species remain in the film) or potential film mass loss if the coordination leads to polymer breakdown. By comparing the mass–charge profiles in dry vs. alcohol-containing electrolytes, we identified the signatures of the proposed degradation mechanism.

## 2. Results and Discussion

In a clean, alcohol-free electrolyte, all investigated Ni–Salen polymer films exhibited stable and reversible redox behavior [21,22]. The oxidation of the Ni(II) centers generates delocalized radical cation states (polarons and bipolarons) along the polymer chains, which are responsible for electronic conduction [22]. Consistent with a p-doping mechanism, a small uptake of anions from the electrolyte is observed upon oxidation to maintain charge neutrality, as evidenced by EQCM mass responses. In a dry electrolyte, the mass–charge curves for these polymers generally exhibit a two-stage behavior: an initial mass gain at the beginning of oxidation due to BF_4_^−^ anion insertion, followed by a plateau, indicating restricted polymer swelling as a result of limited anion insertion [22]. Importantly, in the absence of coordinating impurities, the electrochemical stability of the polymers remains stable over many redox cycles [23].

**Figure 1:**
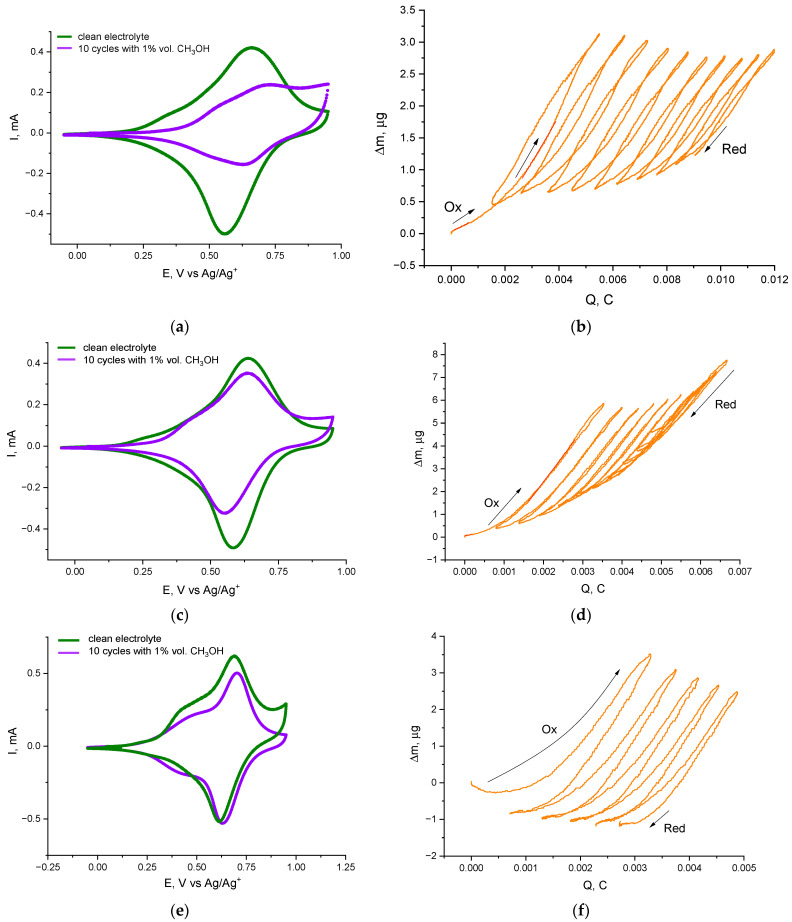
Cyclic voltammograms and EQCM mass–charge responses of Ni Schiff-base polymer films in electrolyte containing 1 vol.% CH_3_OH. (**a**,**b**) poly[Ni(Salen)]; (**c**,**d**) poly[Ni(Salpn-1,2)]; (**e**,**f**) poly[Ni(Saltmen)]. In panels (**a**,**c**,**e**), the green curves correspond to the first cycle in the clean electrolyte, whereas the violet curves show the response after 10 cycles in the presence of CH_3_OH. Panels (**b**,**d**,**f**) present the corresponding EQCM mass change (Δm) as a function of transferred charge (Q); the slope (Δm/ΔQ) provides an effective molar mass associated with charge compensation, and a monotonic net mass increase with cycling indicates irreversible uptake of solvent species within the polymer films.

To evaluate the effect of protic impurities, 1% vol. methanol or isopropanol was introduced into the supporting electrolyte, and the electrochemical behavior of each polymer film was monitored via combined CV and EQCM. Figure 1 summarizes the initial CV (a,c,e) and EQCM Δm vs. time (b,d,f) for each polymer film in the presence of 1% methanol.

It should be acknowledged that the introduction of methanol or isopropanol into the electrolyte may slightly influence ion solvation and overall ionic transport properties of the solution. Protic solvents are capable of interacting with electrolyte species and modifying their local solvation environment. Nevertheless, given the low additive concentration employed in this work (1 vol.%), such effects are expected to be marginal and insufficient to significantly alter the electrochemical response of the system. Therefore, the observed changes in the CV shape and EQCM response are primarily attributed to interactions within the polymer films rather than to bulk electrolyte property changes.

In the methanol-containing electrolyte, all polymer films exhibit a gradual decrease in peak currents and integrated charge with repeated cycling, simultaneously to a monotonic increase in the mass of the films recorded by EQCM. Even by the first few CV cycles, a drop in both anodic and cathodic peak currents is evident for each polymer, though the rate of degradation varies markedly with the complex’s substituents. Notably, the EQCM massograms for cycles in 1% CH_3_OH still show two distinct slopes (two-phase mass uptake) similar to the dry case [24], but with greater total mass gain per cycle. On the first oxidation cycle in methanol, the mass increase for poly[Ni(Salen)] corresponds to an effective molar mass of ~76 g·mol^−1^ per inserted charge (Figure 1a,b), which suggests that each Ni center might be coordinating one methanol molecule along with one acetonitrile co-solvent molecule (methanol: 32 g·mol^−1^, acetonitrile: 41 g·mol^−1^). In contrast, the substituted polymers show a larger initial mass uptake (~220 g·mol^−1^ per charge for poly[Ni(Salpn-1,2)] (Figure 1c,d) and poly[Ni(Saltmen)]) (Figure 1e,f), implying the insertion of one methanol plus approximately four acetonitrile molecules into the more open polymer structure. While this assignment provides a plausible mechanistic interpretation, it should be noted that the EQCM-derived stoichiometries are inherently approximate. The effective molar masses derived from the EQCM mass–charge relationships should be regarded as phenomenological parameters reflecting the dominant mass uptake processes during cycling rather than a unique stoichiometry of coordinated species. Within experimental uncertainty, concurrent contributions such as mixed solvent or ion uptake, partial ligand exchange, or polymer matrix swelling cannot be strictly excluded; therefore, alternative coordination scenarios may also be consistent with the observed EQCM slopes. Importantly, regardless of the exact stoichiometry, the qualitative interpretation remains robust across all investigated polymer systems and electrolyte compositions: monotonic mass accumulation indicates irreversible axial ligand binding, whereas a transition to net mass loss reflects film degradation or detachment. Indeed, all films demonstrate a continuous mass increase over successive cycles in methanol (with no net mass being released back on reduction), indicating irreversible coordination of methanol (and associated solvent) into the polymer matrix: once bound, the methanol is not fully expelled upon reduction, causing cumulative mass growth. It should be noted that in addition to resonance frequency, we monitored the motional (series) resistance R_m_ using the standard QCM200 (Stanford Research Systems) readout, where R_m_ reflects dissipative/viscoelastic loading of the crystal. Under the alcohol-containing conditions used here, the changes in R_m_ remained modest, indicating only a minor contribution of swelling-related viscoelastic effects to the QCM response; therefore, the Sauerbrey-based mass trends discussed below are not dominated by viscoelastic artifacts (although a small contribution cannot be fully excluded).

**Figure 2:**
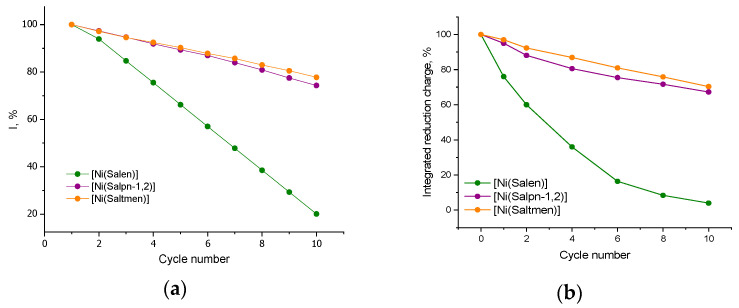
(**a**) Evolution of the anodic peak current and (**b**) evolution of the integrated reduction charge with a cycle number for the poly[Ni(Salen)], poly[Ni(Salpn-1,2)], and poly[Ni(Saltmen)] with the addition of 1% vol. CH_3_OH.

The electrochemical consequences of methanol coordination are clearly reflected in Figure 2, which plots the evolution of the anodic peak current with the cycle number for each polymer in 1% CH_3_OH. The degradation is most pronounced for the unsubstituted poly[Ni(Salen)], which loses ~80% of its peak current after 10 CV cycles in methanol. The EQCM data correlate this rapid capacity loss with a continuous mass increase in the film, supporting that Ni(Salen) complexes are being gradually “poisoned” by methanol ligation and the polymer’s conjugated network is being disrupted. In contrast, the methyl-substituted polymers poly[Ni(Salpn-1,2)] and poly[Ni(Saltmen)] degrade much more slowly in methanol, retaining ~74–76% of their initial peak current after 10 cycles. The enhanced stability of the substituted complexes can be attributed to the steric hindrance provided by the bridge methyl groups, which impedes the axial approach of the methanol molecules to the Ni center. Nevertheless, even these protected polymers show some irreversible capacity loss in methanol, indicating that a fraction of Ni sites still become coordinated.

In the methanol-containing electrolyte (1 vol.% MeOH), the normalized integrated reduction charge decreases monotonically with the cycle number for all polymer films (Figure 2b), indicating a progressive loss of electrochemical activity. The decay is most pronounced for the unsubstituted poly[Ni(Salen)], where the integrated reduction charge drops rapidly over the first few cycles and reaches only a few percentage points of its initial value by the 10th cycle. In contrast, the methyl-substituted polymers poly[Ni(Salpn-1,2)] and poly[Ni(Saltmen)] retain the majority of their charge (≈70% at the 10th cycle), demonstrating substantially higher stability under identical conditions. Importantly, these trends closely mirror the evolution of peak currents observed in the CVs: the strongest decline in peak currents occurs for poly[Ni(Salen)], whereas the substituted polymers exhibit only moderate current decay.

**Figure 3:**
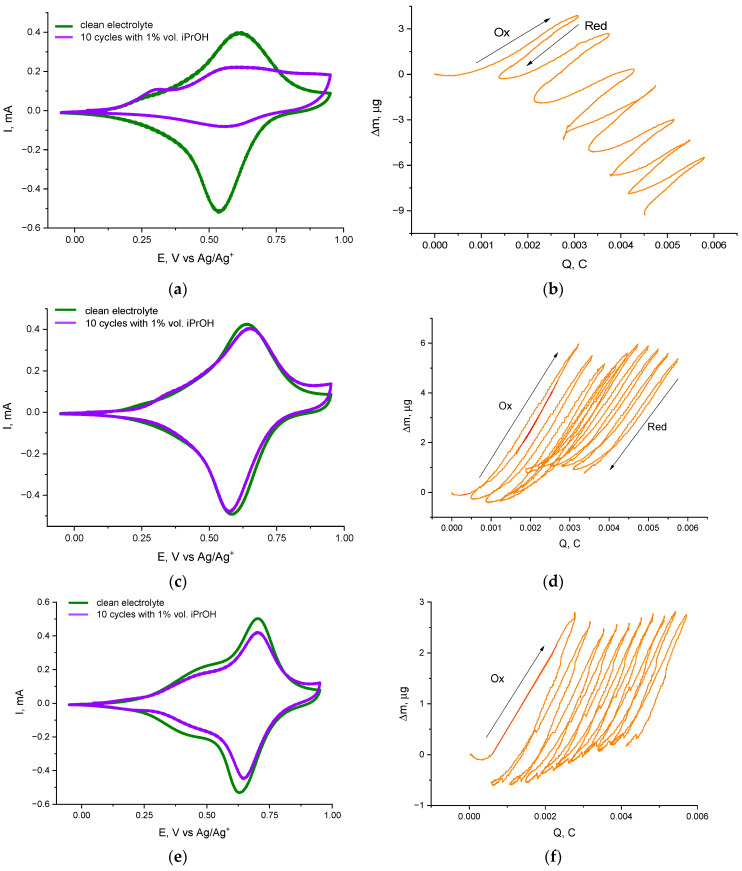
Cyclic voltammograms and EQCM mass–charge responses of Ni Schiff-base polymer films in electrolyte containing 1 vol.% iPrOH. (**a**,**b**) poly[Ni(Salen)]; (**c**,**d**) poly[Ni(Salpn-1,2)]; (**e**,**f**) poly[Ni(Saltmen)]. In panels (**a**,**c**,**e**), the green curves correspond to the first cycle in the clean electrolyte, whereas the violet curves show the response after 10 cycles in the presence of CH_3_OH. Panels (**b**,**d**,**f**) present the corresponding EQCM mass change (Δm) as a function of transferred charge (Q); the slope (Δm/ΔQ) provides an effective molar mass associated with charge compensation, and a monotonic net mass increase with cycling indicates the irreversible uptake of solvent species within the polymer films.

In the case of 1% isopropanol (iPrOH), the divergence in behavior between the unprotected vs. protected polymers becomes even more pronounced. Figure 3a shows that for poly[Ni(Salen)] (no substituents) in iPrOH, the CV peaks deteriorate extremely rapidly—within the first few cycles, the oxidation and reduction peak currents drop sharply. Such a loss of redox reversibility suggests that the poly[Ni(Salen)] polymer is undergoing severe structural degradation or deactivation in the presence of isopropanol. For poly[Ni(Salen)] cycled in an isopropanol-containing electrolyte, the rapid decay of the CV response is accompanied by a transition of the EQCM signal from net mass gain to net mass loss. While the steric bulk of iPrOH is expected to influence axial coordination and may contribute to the destabilization of the polymer structure, the observed mass decrease cannot be uniquely attributed to a single mechanism. In principle, film delamination triggered by swelling-induced mechanical stress could produce an EQCM mass loss; however, purely mechanical detachment would typically be expected to yield a more abrupt mass drop than the gradual, cycle-by-cycle decrease observed here (Figure 3b). The progressive nature of the mass loss therefore suggests that structural degradation is likely accompanied by slow chemical dissolution/erosion of the film (e.g., backbone decomposition or loss of adhesion due to chemical weakening), rather than instantaneous peeling from the electrode. We conclude that the larger size of isopropanol compared to methanol destroys the polymer structure upon coordination. This causes parts of the poly[Ni(Salen)] polymer to break away from the electrode. To verify film integrity, post-cycling inspection of electrode surfaces was performed, confirming the partial loss of film coverage after cycling in the iPrOH-containing electrolyte. Representative post-cycling photographs are shown in Figure S1. The images show that the polymer film is clearly visible on the electrode surface prior to cycling in the isopropanol-containing electrolyte, whereas after cycling, the coating has partially dissolved, being, however, clearly visible as the uniform thinner film on the platinum substrate. Notably, no macroscopic cracks or peeling features are observed, and no visible film fragments or residues are detected in the electrochemical cell, which is consistent with progressive erosion of the film rather than abrupt mechanical delamination.

**Figure 4:**
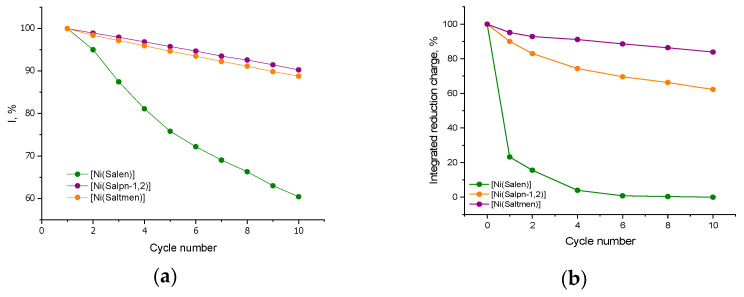
(**a**) Evolution of the anodic peak current and (**b**) evolution of the integrated reduction charge with a cycle number for the poly[Ni(Salen)], poly[Ni(Salpn-1,2)], and poly[Ni(Saltmen)] with the addition of 1% vol. iPrOH.

In contrast, the sterically protected polymers poly[Ni(Salpn-1,2)] and poly[Ni(Saltmen)] demonstrate remarkable resilience in an electrolyte containing isopropanol. As shown in Figure 3c,e, the CV of these substituted polymers remains stable when iPrOH is added. The peak shapes are preserved, and the peak currents diminish only slightly over ten cycles. The presence of the methyl groups on the ligand clearly prevents isopropanol from coordinating in a way that disrupts the redox process. The EQCM data (Figure 3d,f) indicate that both substituted polymers still experience some mass uptake in iPrOH, but significantly, their total film mass remains almost constant over cycling (no significant net gain or loss). Quantitatively, the effective molar masses of inserted species in the first oxidation of substituted polymers were ~230 g·mol^−1^ for poly[Ni(Salpn-1,2)] and ~139 g·mol^−1^ for poly[Ni(Saltmen)]. These values suggest that in poly[Ni(Salpn-1,2)], the Ni sites may coordinate one isopropanol and ~4 acetonitrile molecules, whereas in poly[Ni(Saltmen)], possibly one isopropanol and ~2 solvent molecules enter. The polymer mass changes very little during the next cycles in both cases. The redox activity, although slightly reduced, remains relatively stable—after 10 cycles with 1% iPrOH, poly[Ni(Salpn-1,2)] and poly[Ni(Saltmen)] retain >90% of their initial CV peak currents (less than 10% loss), whereas poly[Ni(Salen)] had lost >60%. Figure 4 compares the degradation rates, showing that the unprotected Ni(Salen) polymer degrades five times faster than the methylated analogs in isopropanol.

In 1 vol.% iPrOH, the integrated reduction charge provides an independent capacity-based metric that supports the stability trends seen in the CVs (Figure 3). The unsubstituted poly[Ni(Salen)] loses charge extremely rapidly, dropping to only a small fraction of its initial value within the first few cycles and remaining at this level thereafter. By contrast, the substituted polymers retain substantially higher charge over repeated cycling, with poly[Ni(Salpn-1,2)] showing the highest retention and poly[Ni(Saltmen)] showing an intermediate behavior. These charge-retention patterns are fully consistent with the corresponding peak-current evolution.

These findings underscore the protective role of steric substitution: by blocking or hindering axial coordination of the larger isopropanol molecules, the substituted polymers avoid catastrophic chain disruption and maintain their electrochemical performance far better.

## 3. Discussion

The distinct mass change behaviors observed (mass accumulation with retained film integrity vs. mass loss with film failure) provide insight into the underlying degradation mechanism. In methanol, all polymers mainly showed mass gain with a retained film structure, consistent with the formation of coordination bonds between Ni centers and methanol. This leads to gradual performance fading but not immediate physical collapse of the film. In isopropanol, the unprotected polymer poly[Ni(Salen)] initially gains mass. This is due to the coordination of iPrOH and solvent. Then, it undergoes mass loss and electrochemical performance collapse. This indicates that the axial ligand (iPrOH) not only coordinates but also causes mechanical stress or bond breakage in the polymer. This is due to its size. As a result, pieces of the film detach. The sterically protected polymers in iPrOH exhibit only minor mass gain without additional changes. This suggests that a limited number of iPrOH molecules can coordinate, and the polymer backbone remains stable. Thus, depending on the polymer structure and impurity, we observe two signatures of the coordination-induced degradation: (1) mass increase with retention of CV shape—indicative of axial ligand binding that disrupts some redox sites but leaves the polymer matrix stable, and (2) mass loss with concurrent drop in electroactivity—indicative of severe polymer damage and material loss, observed for the unprotected polymer. These characteristics strongly support a degradation mechanism dominated by coordination chemistry: external ligand molecules coordinate to the Ni centers, breaking the conjugation and electrical connectivity of the polymer chains, which manifests as a loss of electrochemical activity and, in extreme cases, fragmentation of the polymer film.

Of particular interest is the fact that the behavior of the poly[Ni(Salpn-1,2)] is very close to poly[Ni(Saltmen)] on a quantitative level with both MeOH and iPrOH. This observation means that the one methyl group in the bridge of poly[Ni(Salpn-1,2)] causes virtually the same effect as four methyl groups in the case of poly[Ni(Saltmen)], which allows us to exclude the electronic effects of these substituents as a valuable factor for the reactivity of the polymer towards the alcohols. Moreover, one methyl group in poly[Ni(Salpn-1,2)] might result in the steric protection of only one side of the complex plane, but the resulting protection effect is as strong as the one from four groups from both sides of the complex plane of poly[Ni(Saltmen)], which may explain the coordination behavior of Ni in NiSalen-type complexes. Indeed, the octahedral six-coordinated mode is more favorable for Ni(II) with the Salen ligand rather than the square pyramidal five-coordinated mode. Thus, steric protection of one axial position of the Ni atom disfavors coordination on the opposite axial position, lowering the overall coordination ability of the metal center. Such a behavior contradicts our previous findings on the interaction of sterically hindered NiSalen-type polymers with water, indicating that the interaction mechanism may strongly depend on the nature of the attacking ligand [35].

Overall, the experimental results confirm the universal nature of the axial–ligand degradation model for Ni(Salen) polymers and highlight strategies for mitigation. Both methanol and isopropanol, as protic Lewis bases, can coordinate to Ni in the oxidized polymer, thereby deactivating the redox sites and degrading conductivity. However, molecular design interventions—such as adding steric bulk around the metal—significantly increase the polymer’s tolerance to such impurities. In practical terms, this suggests that by tailoring the ligand structure (e.g., adding blocking groups), one can develop metal–organic polymer electrodes that remain stable even when electrolyte purity is not ideal. This is an important consideration for real energy-storage devices, where trace solvent residues or moisture are often unavoidable.

## 4. Materials and Methods

### 4.1. Materials and Reagents

**Figure 5:**
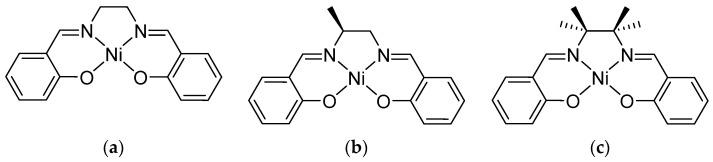
Structures of (**a**) Ni(Salen), (**b**) [Ni(Salpn-1,2)], and (**c**) Ni(Saltmen).

Polymer films of nickel(II) Salen-type complexes were synthesized in situ by anodic electropolymerization of three Ni(II) Schiff-base complex monomers: Ni(Salen), Ni(Salpn-1,2), and Ni(Saltmen). These complexes (Figure 5) share the N,N′-ethylenebis(salicylideneimine) ligand framework (commonly referred to as “Salen”) but differ in substituents: Ni(Salen) is unsubstituted on the diimine bridge, Ni(Salpn-1,2) has two methyl groups on the bridge (1,2-dimethylpropylene diamine backbone), and Ni(Saltmen) has four methyl groups on the bridge (N,N′-tetramethylethylenediamine backbone). Figure 5 illustrates the structures and abbreviations of the monomer complexes: (a) Ni(Salen), (b) Ni(Salpn-1,2), and (c) Ni(Saltmen).

All polymerizations and electrochemical measurements were carried out in an anhydrous acetonitrile (CH_3_CN) solvent (Sigma-Aldrich) containing 0.1 M tetraethylammonium tetrafluoroborate (Et_4_NBF_4_) as the supporting electrolyte. Methanol and isopropanol (HPLC-grade) were used as representative coordinating protic impurities; they were added to the electrolyte at 1% volume concentration.

### 4.2. Cyclic Voltammetry

Polymeric Ni(Salen) films were electrodeposited on glassy carbon (GC) disk electrodes (3 mm diameter, 0.07 cm^2^ area). Electropolymerization was carried out in a CH_3_CN solution containing 1 mM of the respective Ni(II) complex monomer (Ni(Salen), Ni(Salpn-1,2), or Ni(Saltmen)). The electrode potential was held at +0.95 V (vs. Ag/Ag^+^ reference) until a total charge of 0.5 C·cm^−2^ had passed. A platinum wire was used as the counter electrode, and an Ag/Ag^+^ (0.1 M AgNO_3_ in CH_3_CN) electrode calibrated against Ag/AgCl was used as reference. After polymerization, the film-coated electrodes were rinsed with dry acetonitrile to remove any monomer or oligomer residues.

CV was conducted in a sealed three-electrode cell under an argon atmosphere (to prevent moisture ingress). The electrolyte was the dried 0.1 M Et_4_NBF_4_/CH_3_CN solution, either neat (dry electrolyte) or spiked with 1% vol. of methanol or isopropanol. The working electrode was the polymer-coated GC disk. The CV scans were performed using an Autolab PGSTAT30 potentiostat (Metrohm Autolab B.V., Utrecht, The Netherlands) over a potential window from—0.1 V to +0.95 V vs. Ag/Ag^+^, at a sweep rate of 50 mV·s^−1^. Each experiment involved 10–50 cyclic potential sweeps; unless otherwise noted, results are reported for 10 cycles. The first cycle was used to stabilize the film in the new electrolyte, and subsequent cycles were analyzed for degradation effects. The capacitance (charge storage) of the polymer films was evaluated from the integrated charge under the oxidation/reduction peaks in the CV. Film stability was quantified by the retention of integrated redox charge (or peak current) after repeated cycling: the decrease in anodic peak charge after 10 cycles (as a percentage of the initial cycle) was used as a measure of electrochemical stability in each condition.

### 4.3. Electrochemical Quartz Crystal Microbalance

EQCM measurements were performed using a QCM200 quartz microbalance system (Stanford Research Systems, Sunnyvale, CA, USA) equipped with 5 MHz AT-cut quartz crystals coated with Ti/Pt (active area: 1.37 cm^2^). For EQCM measurements, polymer films were grown under identical conditions on AT-cut quartz crystal resonators (coated with thin-film platinum electrodes). The electropolymerization on the quartz working electrode used the same potential (+0.95 V vs. Ag/Ag^+^) and charge limit (0.5 C·cm^−2^) as for the GC electrodes. After deposition, the quartz crystals with polymer films were thoroughly rinsed with dry CH_3_CN and transferred to the EQCM cell.

EQCM measurements were performed simultaneously with cyclic voltammetry to monitor mass changes in the polymer films during redox cycling. A 9 MHz quartz crystal with a Pt electrode (area 0.2 cm^2^) coated by the polymer served as the EQCM working electrode. The EQCM cell was hermetically sealed and purged with argon throughout the experiment. The same potential program was applied as described for CV (−0.1 V to +0.95 V vs. Ag/Ag^+^, 50 mV·s^−1^, 10 cycles). The resonant frequency of the quartz was recorded in real time and converted to mass change (Δm) using the Sauerbrey equation [20], assuming rigid mass attachment. A positive Δm corresponds to mass uptake by the film (e.g., due to insertion of electrolyte ions/solvent), whereas a negative Δm indicates mass loss (e.g., expulsion of species or film detachment).

## 5. Conclusions

This study demonstrated that the stability of Ni(Salen) polymer in an organic electrolyte is highly dependent on its molecular structure, especially in the presence of coordinating impurities. In a clean electrolyte, all studied polymer films (unsubstituted and substituted) exhibited stable and reversible electrochemical behavior. However, the addition of just 1% vol. of monohydric alcohols (methanol or isopropanol) to the electrolyte induced significant degradation of the electrochemical response, most notably for the unsubstituted poly[Ni(Salen)] complex. Films of poly[Ni(Salen)] showed the greatest susceptibility to performance loss in the alcohol-containing electrolytes, whereas introducing methyl substituents into the diimine bridge of the ligand (as in poly[Ni(Salpn-1,2)] and poly[Ni(Saltmen)]) led to a dramatic improvement in stability.

By utilizing cyclic voltammetry with EQCM, we identified distinguishing features of the degradation mechanism. Depending on the polymer and impurity, the films exhibited either (a) continuous mass increase during cycling (with the CV peak shape largely preserved) or (b) mass loss accompanied by a concurrent drop in electrochemical activity. These observations are consistent with a coordination-induced degradation mechanism: external ligand molecules (here, alcohol impurities) coordinate axially to the Ni center in the oxidized state, which interrupts the conjugated pathway of the polymer and diminishes its redox activity. In cases of mild coordination (e.g., methanol or isopropanol with protected polymers), the polymer experiences mass uptake and gradual capacity fade; in more severe cases (isopropanol with unprotected Ni(Salen)), coordination leads to polymer chain scission or detachment, evidenced by mass loss and abrupt performance failure.

The results underscore the universality of the axial–ligand degradation model for this class of materials and, importantly, highlight the critical role of steric protection in enhancing polymer stability. Providing steric hindrance around the metal center effectively limits the access of deleterious impurities, thereby safeguarding the polymer’s electrochemical function. These findings offer valuable insights for the molecular design of robust electroactive polymers. By incorporating appropriate ligand substitutions, it is possible to create efficient and stable materials for organic energy-storage devices even under less-than-ideal electrolyte conditions. These principles may be extended to the sustainable design of a large variety of functional materials based on coordination polymers and MSalen-type molecules, making it more resistant to the degradation promoted by various small-molecule contaminants. This work opens up broad possibilities for targeted molecular engineering of new metallopolymers aimed at high performance and durability in batteries, supercapacitors, and other electrochemical energy technologies.

## Data Availability

The original contributions presented in this study are included in the article/Supplementary Material. Further inquiries can be directed to the corresponding author.

## Supplementary Materials

The following supporting information can be downloaded at: https://www.mdpi.com/article/10.3390/ijms27041685/s1.

## Abbreviations

The following abbreviations are used in this manuscript:

CV	Cyclic voltammetry
EQCM	Electrochemical quartz crystal microbalance
iPrOH	Isopropanole
GC	Glassy carbon
