# Supplementary material for: Effect of Coordinating Impurities on the Electrochemical Stability of Polymeric Nickel(II) Schiff-Base Complexes
Source: Int J Mol Sci. 2026 Feb 9;27(4):1685. doi: 10.3390/ijms27041685 (PMC12940595; doi:10.3390/ijms27041685)
Supplement: Supplementary file 1 [file ijms-27-01685-s001.zip › ijms-4100966-supplementary.pdf]

*Article*

# Effect of Coordinating Impurities on the Electrochemical Stability of Polymeric Nickel(II) Schiff-Base Complexes

**Ulyana M. Rodionova [1], Daniil A. Lukyanov [1], Peixia Yang [2], Ruopeng Li [2], Oleg V. Levin [1] and Elena V. Alekseeva [1,*]**

[1] Institute of Chemistry, Saint-Petersburg University, 199034 Saint-Petersburg, Russia
[2] Harbin Institute of Technology, Harbin 150001, China
* Correspondence: e.v.alekseeva@spbu.ru

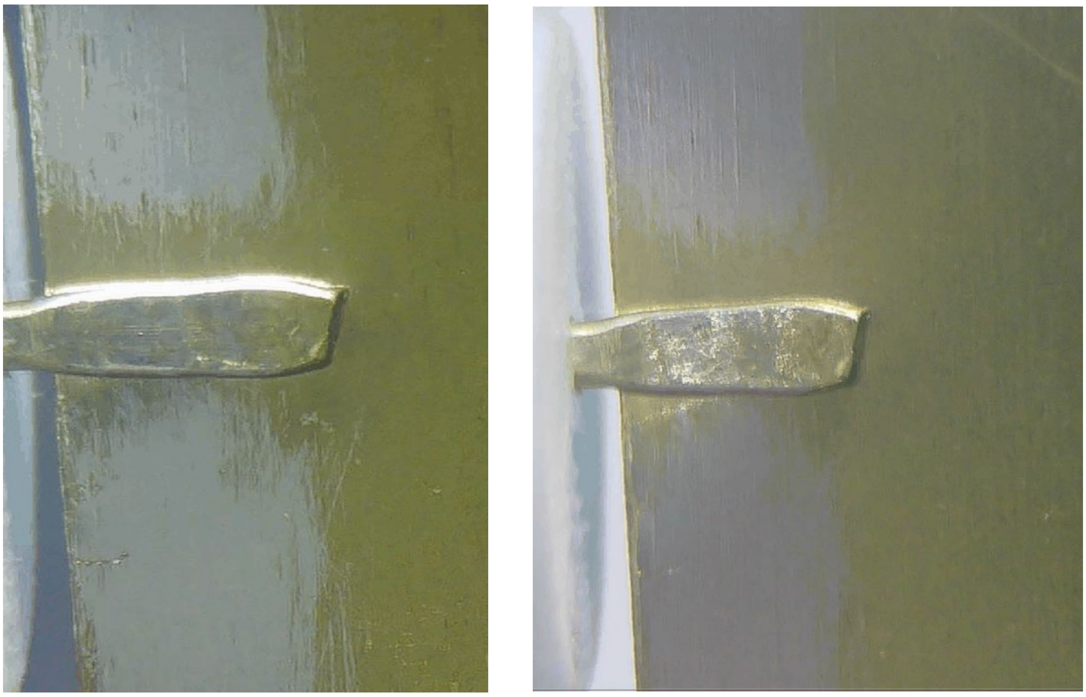

(a) (b)

Figure S1. Optical photographs of a poly[Ni(Salen)]-coated electrode before (a) and after (b) electrochemical cycling in electrolyte containing 1 vol.% isopropanol (iPrOH).
